# Pseudogout Mimicking Aortic Dissection: A Case Report

**DOI:** 10.7759/cureus.6239

**Published:** 2019-11-26

**Authors:** Kaylah D Maloney, Arjun Balakumar, Sean McGann, Xiao Chi Zhang

**Affiliations:** 1 Emergency Medicine, Thomas Jefferson University Hospital, Philadelphia, USA; 2 Emergency Medicine, Sidney Kimmel Medical College at Thomas Jefferson University, Philadelphia, USA

**Keywords:** pseudogout, adult, emergency medicine, rheumatology, radiographic studies, treatment

## Abstract

Calcium pyrophosphate dihydrate (CPPD) crystal deposition disease or pseudogout is an idiopathic articular disease that predominantly affects elderly patients. It is caused by a systemic deposition of calcium pyrophosphate (CPP) crystals in the articular and hyaline joint cartilage. The majority of cases present as chronic arthritis, but a subset of CPPD can present as rapid onset of sharp pain and joint swelling, posing a diagnostic challenge. We present a case of a 64-year-old man with a history of hypertension, urologic cancer, and gout presenting to the emergency department (ED) with a sudden-onset, severe stabbing right shoulder pain radiating to the neck and upper back. On ED arrival, he was mildly hypotensive, afebrile, diaphoretic, and uncomfortable, causing concern for aortic dissection. His exam was significant for limited shoulder range of motion; his sensation, strength, and distal pulses were intact and equal in bilateral upper extremities. His plain films showed multilevel cervical degenerative disc disease and facet arthrosis and right glenohumeral osteoarthritis without fracture or malalignment. A computed tomography (CT) angiogram was negative for vascular anomalies. Throughout his ED stay, his pain was refractory to medication, and he developed a new fever, prompting a targeted shoulder ultrasound; this revealed large glenohumeral effusion, and synovial analysis revealed CPP crystals without organism growth. This case illustrates an unusual acute CPPD attack that mimicked an aortic dissection. Emergency physicians should recognize both common and uncommon presentations for chronic disease processes in maintaining a broad differential diagnosis and delivering quick, targeted treatment.

## Introduction

Calcium pyrophosphate dihydrate (CPPD) crystal deposition disease or pseudogout results from an imbalance of calcium pyrophosphate (CPP) crystals with deposition in the articular and hyaline cartilage of joints [1]. CPPD occurs in 4%-7% of the US population with prevalence increasing with age [2-4]. Although the majority present as chronic arthritis, a subset of CPPD can present as rapid onset of sharp pain and joint swelling, mimicking diseases such as septic arthritis, intra-articular hemorrhage, and vascular dissection.

## Case presentation

A 64-year-old man presented to the emergency department (ED) with a sudden-onset, severe stabbing right shoulder pain radiating to the neck and back. The symptoms began five days earlier and were exacerbated with palpation and range of motion. His medical history included sarcoidosis, hypertension, prostate cancer, bladder tumor, and gout. His medications included prednisone, cellcept, nimodipine, aspirin, and ranitidine. Review of system was positive for numbness, tingling and weakness of his right arm, negative for trauma, fevers, chills, chest pain, and shortness of breath. On arrival, the patient’s vital signs were blood pressure of 86/60 mmHg, heart rate of 70 beats per minute, respiratory rate of 20 breaths per minute, temperature of 97.7°F, and a SaO2 of 98% on room air. His physical exam was noticeable for an uncomfortable-appearing, diaphoretic male with limited right shoulder range of motion secondary to pain; his sensation, strength, and distal pulses were intact and equal in bilateral upper extremities. His skin exam was without erythema or warmth over the right shoulder joint.

**Figure 1:**
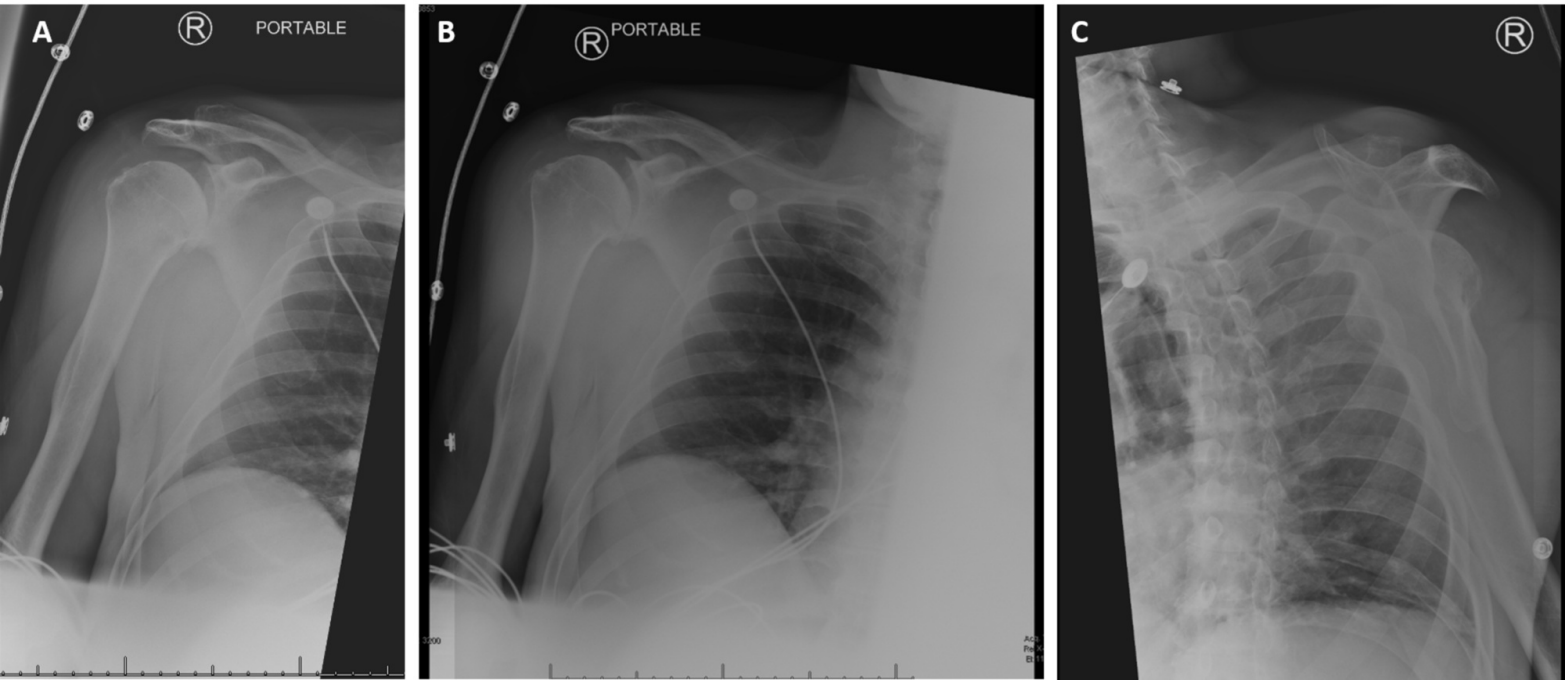
Right shoulder X-ray of the patient

Initial laboratory results were significant for a white blood count of 8.5 B/L (reference range: 4-11 B/L), C-reactive protein of 1.2 (reference range < 0.8 mg/dL), sedimentation rate of 9 (reference range: 0-20), high-sensitivity troponins 12 (reference range <19 mg/L). An electrocardiogram showed an unchanged left anterior fascicular block. His chest plain-film and cervical spine plain-film showed multilevel degenerative disc disease and facet arthrosis. His right shoulder plain film demonstrated glenohumeral osteoarthritis without fracture or malalignment (Figure 1). A bedside echocardiogram showed no evidence of pericardial effusion and his computed tomography (CT) angiogram was negative for aortic dissection, aneurysm or intramural hematoma.

**Figure 2:**
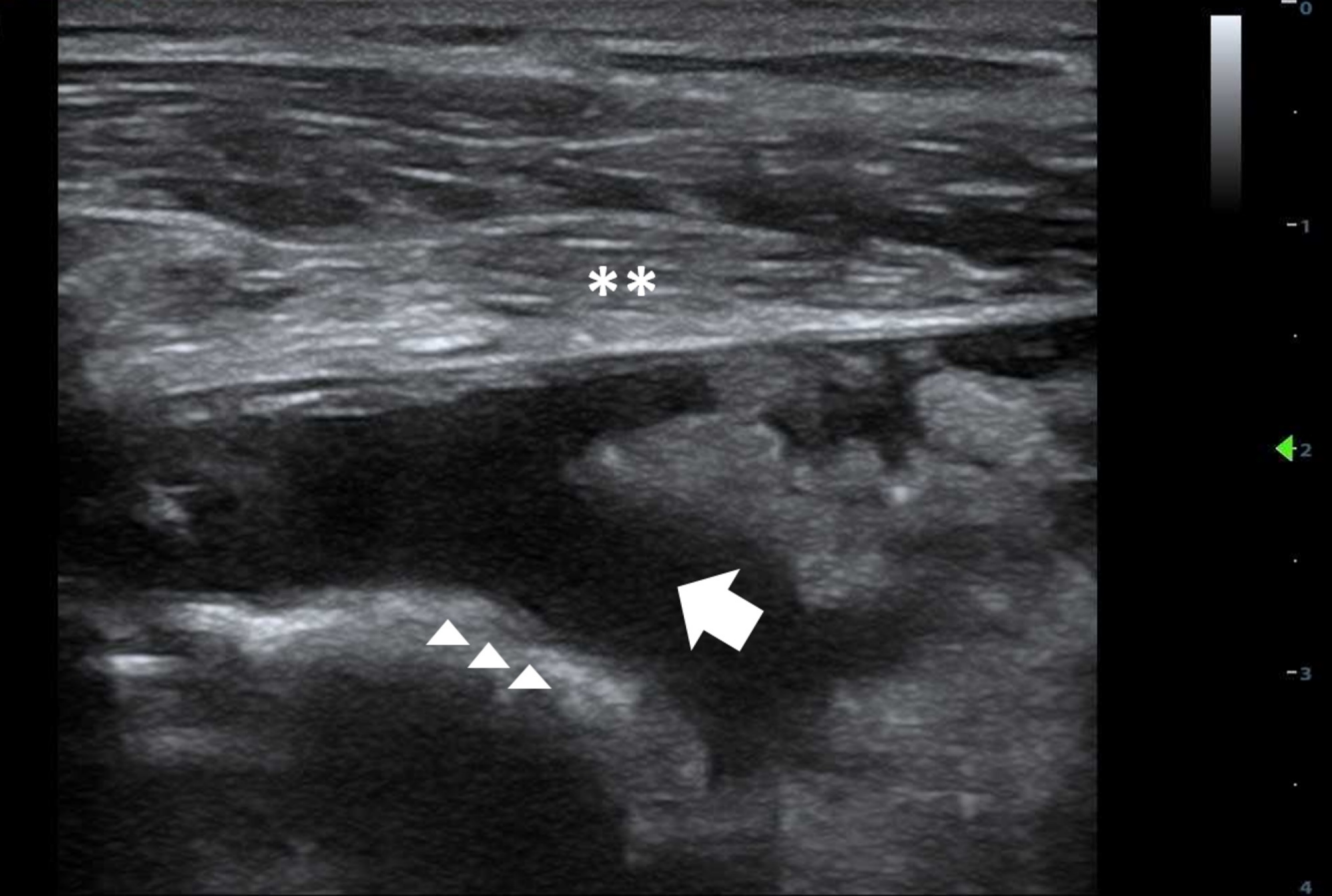
Right shoulder ultrasound showing fluid collection **- deltoid ^^^- humeral head

The patient continued to endorse severe pain refractory to intravenous analgesia and he subsequently developed a fever of 100.6°F, prompting a point-of-care (POCUS) shoulder ultrasound that revealed large glenohumeral effusion (Figure 2). Synovial analysis revealed 688 white blood cells with 96% neutrophils (reference range < 150 /uL); 5,669 red blood cells (reference range: <0 /uL); and positive birefringence crystals consistent with CPP without evidence of organisms. The patient was diagnosed with acute pseudogout flare and admitted for pain control.

## Discussion

CPPD traditionally presents as a progressive disease with subacute pain and inflammation developing in the knee or wrist, however, acute CPPD attacks may also occur after high stressors such as surgery or trauma [5-6]. These acute attacks can be associated with fever, malaise, and radiating pain that persist for weeks [7-8]. While most acute CPPD attacks are self-limited, intervention may prevent serious chronic complications such as joint degeneration, spinal stenosis, and crowded dens after repetitive attacks [9-10].

CPPD is diagnosed by the presence of synovial rhomboid-shaped, positively birefringent crystals or appearance of heavy-rounded fibrocartilage calcifications on plain-film. POCUS can also distinguish CPP from monosodium urate (MSU) crystals, as MSU appear on the cartilage surface, whereas CPP crystals form within joint cartilage [11]. As such, treatment is aimed to manage the crystal-induced inflammation. Traditional medications including glucocorticoids and non-steroidal anti-inflammatories are first-line treatments for acute presentation [12]. Second-line agents include adrenocorticotropic hormone (ACTH) and colchicine for sub-acute presentations, although only case reports have shown efficacy with ACTH in significant symptom attenuation [13]. Colchicine should be considered only for long-term management and avoided in acute attacks due to its significant side-effect profile [14].

Our patient’s diagnosis was confirmed with a POCUS study and arthrocentesis with joint fluid analysis in the setting of refractory pain and new-onset fever that was missed on initial presentation. This case illustrates that pseudogout can be easily misdiagnosed due to high sub-clinical prevalence and reliance on traditional imaging modalities. Risk-factor identification in elderly patients presenting with acute monoarticular inflammation should prompt clinicians to consider emergent joint pathologies and recognize limitations in diagnosing pseudogout with traditional plain film or CT imaging. While other conditions may pose a more serious threat to survival and should be thoroughly evaluated on top of a chronic condition such as pseudogout, the cause of underlying pain and discomfort should not be ignored. 

## Conclusions

Emergency physicians should remain vigilant in recognizing common and uncommon chronic disease presentations to deliver quick, targeted treatment. Pseudogout, CPPD, is a chronic joint disorder that can mimic severe disease processes such as septic arthritis or aortic dissection during acute exacerbation in the setting of shock trauma or recent stressors. Clinicians should utilize POCUS to look for joint pathologies on patients presenting with unilateral chest discomforts, as joint effusion may be missed on both traditional and advanced radiographic imaging.

## References

[1] Sidari A, Hill E (2018). Diagnosis and treatment of gout and pseudogout for everyday practice. Prim Care.

[2] Felson DT, Naimark A, Anderson J, Kazis L, Castelli W, Meenan RF (1987). The prevalence of knee osteoarthritis in the elderly. The framingham osteoarthritis study. Arthritis Rheum.

[3] Rosenthal AK, Ryan LM (2016). Calcium pyrophosphate deposition disease. N Engl J Med.

[4] Rosenthal AK (2006). Pseudogout: Presentation, natural history, and associated conditions. Crystal-Induced Arthropathies: Gout, Pseudogout, and Apatite-Associated Syndromes.

[5] Joseph J, McGrath H (1995). Gout or “pseudogout”: how to differentiate crystal-induced arthropathies. Geriatrics.

[6] Dieppe PA, Alexander GJ, Jones HE, Doherty M, Scott DGI, Manhire A, Watt I (1982). Pyrophosphate arthropathy: a clinical and radiological study of 105 cases. Ann Rheum Dis.

[7] Miksanek J, Rosenthal AK (2015). Imaging of calcium pyrophosphate deposition disease. Curr Rheumatol Rep.

[8] Andres M, Sivera F, Pascual E (2018). Therapy for CPPD: options and evidence. Curr Rheumatol Rep.

[9] Gerster JC, Vischer TL, Fallet GH (1975). Destructive arthropathy in generalized osteoarthritis with articular chondrocalcinosis. J Rheumatol.

[10] Rosenthal AK, Ryan LM (2011). Calcium pyrophosphate deposition-nothing 'pseudo' about it!. Nat Rev Rheumatol.

[11] Grassi W, Meenagh G, Pascual E, Filippucci E (2006). "Crystal clear"-sonographic assessment of gout and calcium pyrophosphate deposition disease. Semin Arthritis Rheum.

[12] Barrack RL, Jennings RW, Wolfe MW, Bertot AJ (1997). The Coventry award: the value of preoperative aspiration before total knee revision. Clin Orthop Relat Res.

[13] Daoussis D, Antonopoulos I, Yiannopoulos G, Andonopoulos AP (2014). ACTH as first line treatment for acute calcium pyrophosphate crystal arthritis in 14 hospitalized patients. Joint Bone Spine.

[14] Zhang W, Doherty M, Bardin T (2011). European League Against Rheumatism recommendations for calcium pyrophosphate deposition. Part I: terminology and diagnosis. Ann Rheum Dis.

